# Association of advanced paternal age with lung function at school age

**DOI:** 10.1186/s12931-022-02178-4

**Published:** 2022-09-20

**Authors:** Chun-Chun Gau, Hsin-Ju Lee, Hung-Yi Lu, Chao-Yi Wu, Hsin-Yi Huang, Hui-Ju Tsai, Tsung-Chieh Yao

**Affiliations:** 1 Division of Allergy, Asthma, and Rheumatology, Department of Pediatrics, Chang Gung Memorial Hospital, 5 Fu-Hsin Street, Kweishan, Taoyuan, Taiwan; 2 School of Medicine, Chang Gung University College of Medicine, Taoyuan, Taiwan; 3 Division of Pediatric General Medicine, Department of Pediatrics, Chang Gung Memorial Hospital, Taoyuan, Taiwan; 4 Department of Pediatrics, New Taipei Municipal TuCheng Hospital, New Taipei, Taiwan; 5 Institute of Population Health Sciences, National Health Research Institutes, 35 Keyan Road, Zhunan, Miaoli County, 35053 Taiwan

**Keywords:** Paternal age, Lung function, Children, Environmental tobacco smoke, Breastfeeding

## Abstract

**Background:**

Epidemiological studies suggest that advanced paternal age impact offspring health, but its impact on respiratory health is unclear. This study aimed to investigate the association of paternal age with lung function and fraction of exhaled nitric oxide (FeNO) in children.

**Methods:**

We analyzed data from 1330 single-born children (576 girls, 43.3%; mean age, 6.4 years), who participated in the Longitudinal Investigation of Global Health in Taiwanese Schoolchildren (LIGHTS) cohort and received measurements of lung function and FeNO at 6-year follow-up visits. Covariate-adjusted regression analyses were applied.

**Results:**

Every 5-year increase in paternal age at birth was associated with 0.51% decrease in FEV_1_/FVC ratio (95% CI − 0.86 to − 0.15; *p* = 0.005) and 19.86 mL/s decrease in FEF_75_ (95% CI: − 34.07 to − 5.65; *p* = 0.006). Stratified analyses revealed that increasing paternal age at birth was associated with decreasing FEV_1_/FVC ratio and FEF_75_ only among children with prenatal exposure to environmental tobacco smoke (ETS) or not being breastfed. Sensitivity analyses using paternal age as a categorical variable found decreasing FEV_1_/FVC ratio and FEF_75_ in the groups of paternal age 35–39 and ≥ 40 years. There was no association of paternal age at birth with FeNO.

**Conclusion:**

Our findings provide novel evidence linking advanced paternal age at birth with decreasing lung function in children at school age. Children with prenatal exposure to ETS or not being breastfed are more vulnerable to the adverse effect of advanced paternal age on childhood lung function. Further studies are warranted to confirm this novel adverse effect of advanced paternal age.

**Supplementary Information:**

The online version contains supplementary material available at 10.1186/s12931-022-02178-4.

## Introduction

There has been a shift toward delayed childbearing in many countries over the past few decades [1–3]. Several studies have reported that advanced paternal age is associated with increased risks of chromosomal and non-chromosomal birth defects and neuropsychiatric disorders including epilepsy, schizophrenia, and autism [4–6]. The mechanisms behind the harmful effects of advanced paternal age on offspring’s health remain unclear, but may be related to *de novo* mutations, epigenetic changes, and DNA damage accumulations in male germ cells [7]. Another possible explanation is increasing paternal age may be responsible for the accumulation of exposure to environmental hazards, e.g., tobacco smoke and alcohol consumption [8, 9].

Previous studies have mainly reported association of parental age with birth defects and negative neuropsychiatric outcomes [4–6]. Two European studies have suggested that advanced paternal age may be associated with a decreased risk of asthma in childhood [10, 11]. Still, limited studies have evaluated advanced parental age at delivery in relation to long-term consequences for respiratory health in offspring [12]. In a population-based survey, Gomez Real et al. suggested that advanced maternal age at delivery was associated with higher lung function in offspring [12]. However, the impact of advanced paternal age at birth on respiratory health in offspring remains largely unknown. The relationship of paternal age with lung function, an objective measure of general respiratory health [13, 14], and the fraction of exhaled nitric oxide (FeNO), a noninvasive biomarker of airway inflammation [15], has not yet been studied, which may shed light on the impact of advanced paternal age on respiratory health.

In this large prospective population-based cohort study, we aimed to investigate whether paternal age at birth is associated with lung function and FeNO in children at school age, and to determine potential effect modifiers, specifically, prenatal exposure to environmental tobacco smoke (ETS) and whether being breastfed during the first six months.

## Methods

### Subjects

**Fig. 1:**
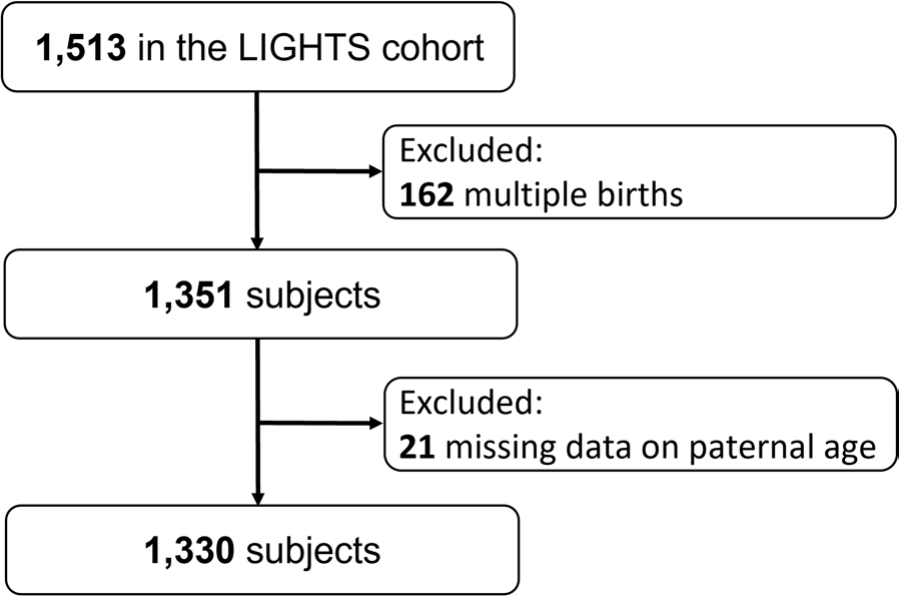
Recruitment process of the study participants. *LIGHTS* Longitudinal Investigation of Global Health in Taiwanese Schoolchildren

This study is a part of the Longitudinal Investigation of Global Health in Taiwanese Schoolchildren (LIGHTS) study, which is a population-based longitudinal cohort included 1513 children born during 2010–2011 in the Chang Gung Memorial Hospital [16–19]. The majority of these children lived in northwestern Taiwan. Demographic, epidemiological and clinical data of study participants were collected using a questionnaire, anthropometry, spirometry, and FeNO measurement at a 6-year follow-up visit during 2016–2018. We obtained the perinatal information from electronic medical records in the Chang Gung Memorial Hospital. A questionnaire was fulfilled by parents of study participants to collect the information of socio-demographics, physician-diagnosed asthma, parental allergic diseases, prenatal exposure to ETS, and breastfeeding. All participants had their heights measured according to a standard protocol. A total of 1330 single-born children were included in this study after exclusion of multiple births (n = 162) and missing data on the paternal age (n = 21) (Fig. 1). This study was approved by the Institutional Review Board of Chang Gung Medical Foundation (IRB 201600334A3). Parents of each participant provided written informed consents.

### Paternal age at birth

Father’s date of birth was obtained from the father’s identity documents. Paternal age at birth was calculated based on the date of the child’s birth. Paternal age at birth was treated as a continuous variable or as a categorical variable with four groups, including < 30 (reference group), 30–34, 35–39, and ≥ 40 years, which were used for subsequent analyses.

### Lung function and FeNO

Lung function was measured by spirometry (Spirolab II, Medical International Research, Italy) according to the American Thoracic Society (ATS)/ European Respiratory Society (ERS) recommendations [20]. Lung function parameters including forced vital capacity (FVC), forced expiratory volume in 1 s (FEV_1_), FEV_1_/FVC ratio, forced expiratory flow at 75% of FVC (FEF_75_), and peak expiratory flow (PEF) were recorded. FVC, FEV_1_, FEV_1_/FVC ratio, and FEF_75_ were also converted to *z*-scores based on the Global Lung Function Initiative (GLI) 2012 South East Asian equations [21]. FeNO was measured in parts per billion (ppb) using the chemiluminescence analyzer (CLD 88sp NO analyzer®, Eco Medics, Switerland) in accordance with the 2005 ATS/ERS recommendations [22].

### Statistical analysis

We used multiple linear regression models to examine the associations between paternal age at birth and outcomes of interest, including lung function parameters (FVC, FEV_1_, FEV_1_/FVC ratio, FEF_75_, and PEF) and FeNO, with adjustment of relevant covariates. Paternal age at birth was analyzed either as a continuous variable or as a categorical variable with four groups, including < 30 (reference group), 30–34, 35–39, and ≥ 40 years. The adjusted covariates were listed as follows: child’s age, sex, height, maternal age at birth, prematurity (gestational age less than 37 weeks), birth weight, cesarean delivery, birth order, physician-diagnosed asthma, parental university education, parental allergic diseases (physician-diagnosed asthma, allergic rhinitis or atopic dermatitis in mother, father or both), prenatal exposure to ETS (one or more household smokers during gestation), breastfeeding (exclusive or partial) longer than 6 months, and household income, which were similar to previous relevant studies [12, 14, 17, 23]. We performed subgroup analysis, stratified by prenatal exposure to ETS and breastfeeding which were associated with lung function and FeNO in previous studies [24–29], to evaluate potential effect modifiers. Sensitivity analyses were performed by converting lung function parameters to *z*-scores based on the GLI-2012 reference equations. Statistical analysis was performed using the SPSS Statistics version 22.0 (SPSS Inc., Armonk, NY, United States). A *p*-value less than 0.05 was considered statistically significant.

## Results

Table 1 shows the characteristics of the study subjects. Among 1330 children (576 girls, 43.3%), the mean age was 6.4 years (standard deviation [SD]: 0.4). The mean paternal age at birth was 34.7 years (SD: 5.3). Lung function and FeNO were successfully measured in 1322 and 1275 children, respectively.

**Table 1:** Characteristics of study participants

Characteristic	*N*	Data
Age, mean ± SD (year)	1330	6.4 ± 0.4
Sex, female, *n* (%)	1330	576 (43.3%)
Height, mean ± SD (cm)	1330	118.5 ± 5.7
Paternal age at birth, mean ± SD (year)	1330	34.7 ± 5.3
Maternal age at birth, mean ± SD (year)	1330	32.2 ± 4.1
Prematurity, *n* (%)	1330	204 (15.3%)
Birth weight, mean ± SD (gm)	1330	2,999.2 ± 615.6
Cesarean delivery, *n* (%)	1330	473 (35.6%)
Birth order, *n* (%)	1329	
First		742 (55.8%)
Second		475 (35.8%)
Third or later		112 (8.4%)
Physician-diagnosed asthma	1323	305 (23.1)
Parental university education, *n* (%)	1330	1,164 (87.5%)
Parental allergic diseases, *n* (%)	1324	932 (70.4%)
Prenatal exposure to ETS, *n* (%)	1300	541 (41.6%)
Breastfeeding, *n* (%)	1328	611 (46.0%)
Household income per year, *n* (%)	1321	
< 300,000 NTD		42 (3.2%)
300,000-600,000 NTD		216 (16.4%)
600,000-900,000 NTD		290 (22.0%)
900,000–1,200,000 NTD		353 (26.7%)
> 1,200,000 NTD		420 (31.8%)
Lung function, mean ± SD	1322	
FVC (mL)		1192.4 ± 236.5
FEV_1_ (mL)		1091.7 ± 216.4
FEV_1_/FVC ratio (%)		91.7 ± 6.3
FEF_75_ (mL/s)		848.2 ± 268.7
PEF (mL/s)		2070.5 ± 620.7
FeNO (ppb), mean ± SD	1275	32.2 ± 4.1

*n* number, *SD* standard deviation SD, *ETS* environmental tobacco smoke, *NTD* New Taiwan Dollar, *FVC* forced vital capacity *FEV*_1_ forced expiratory volume in 1 s, *FEF*_75_ forced expiratory flow at 75% of FVC, *PEF* peak expiratory flow, *ppb* parts per billion

Increasing paternal age at birth was significantly associated with decreasing FEV_1_/FVC ratio and FEF_75_, after adjustment for child’s age, sex, height, maternal age at birth, prematurity, birth weight, cesarean delivery, birth order, physician-diagnosed asthma, parental university education, parental allergic diseases, prenatal exposure to ETS, breastfeeding, and household income (Table 2). Specifically, every 5-year increase in paternal age at birth was associated with 0.51% decrease in FEV_1_/FVC (95% CI: − 0.86 to − 0.15; *p* = 0.005) and 19.86 mL/s decrease in FEF_75_ (95% CI: − 34.07 to − 5.65; *p* = 0.006). There were no associations of paternal age at birth with FVC, FEV_1_, PEF, or FeNO (Table 2). Similar results were found when we performed sensitivity analyses by converting lung function parameters to *z*-scores (Additional file 1: Table S1).

**Table 2:** Association between paternal age at birth (continuous, per 5-year increase) and lung function

	Crude coefficient β (95% CI)^a^	Adjusted coefficient β (95% CI)^a^
FVC (mL)	− 1.32 (− 13.33, 10.69)	− 0.45 (− 9.72, 8.82)
FEV_1_ (mL)	− 6.15 (− 17.14, 4.83)	− 5.95 (− 14.55, 2.65)
FEV_1_/FVC (%)	− 0.42 (− 0.74, − 0.10)^*^	− 0.51 (− 0.86, − 0.15)^*^
FEF_75_ (mL/s)	− 20.11 (− 33.73, − 6.49)^*^	− 19.86 (− 34.07, − 5.65)^*^
PEF (mL/s)	− 19.49 (− 50.84, 11.86)	− 16.61 (− 47.13, 13.91)
FeNO (ppb)	− 0.06 (− 0.97, 0.85)	− 0.35 (− 1.36, 0.65)

*CI* confidence interval, *FVC* forced vital capacity,* FEV*_1_ forced expiratory volume in 1 s,* FEF*_75_ forced expiratory flow at 75% of FVC,* PEF* peak expiratory flow; ppb: parts per billion

^a^ Per 5-year increase in paternal age at birth, adjusting for age, sex, height, maternal age at birth, prematurity, birth weight, cesarean delivery, birth order, physician-diagnosed asthma, parental university education, parental allergic diseases, prenatal exposure to environmental tobacco smoke, breastfeeding, and household income

^*^ *P* < 0.05 is bold

Stratified analyses were conducted to assess whether the effects of paternal age at birth on lung function is modified by prenatal exposure to ETS or breastfeeding. When stratified by prenatal exposure to ETS, increasing paternal age at birth was significantly associated with decreasing FEV_1_, FEV_1_/FVC ratio, FEF_75_, and PEF after adjustments of relevant confounders only among children with prenatal exposure to ETS, but not among the non-exposure counterparts (Table 3). Among the children with prenatal exposure to ETS, every 5-year increase in paternal age at birth was associated with 15.11 mL decrease in FEV_1_ (95% CI: − 29.06 to − 1.16; *p* = 0.034), 0.77% decrease in FEV_1_/FVC (95% CI: − 1.34 to − 0.20; *p* = 0.008), 27.21 mL/s decrease in FEF_75_ (95% CI: − 49.28 to − 5.15; *p* = 0.016), and 50.95 mL/s decrease in PEF (− 99.81 to − 2.09; *p* = 0.041). When stratified by breastfeeding, the negative association between paternal age at birth and lung function was statistically significant only in children not being breastfed or breastfed less than 6 months, but not in those being breasted longer than 6 months (Table 4). Among children not being breastfed or breastfed less than 6 months, every five-year increase in paternal age at birth was associated with 0.60% decrease in FEV_1_/FVC (95% CI: − 1.04 to − 0.15; *p* = 0.009) and 22.31 mL decrease in FEF_75_ (95% CI: − 40.75 to − 3.87; *p* = 0.018).

**Table 3:** Association between paternal age (continuous, per 5-year increase) and lung function, stratified by prenatal exposure to environmental tobacco smoke

	Crude coefficient β (95% CI)^a^	Adjusted coefficient β (95% CI)^a^
Prenatal exposure to ETS ( n = 541)		
FVC (mL)	− 4.19 (− 22.97, 14.59)	− 7.39 (− 22.59, 7.81)
FEV_1_ (mL)	− 11.60 (− 28.58, 5.37)	− 15.11 (− 29.06, − 1.16)^*^
FEV_1_/FVC (%)	**− 0.64 (− 1.14, − 0.13)** ^*^	− 0.77 (− 1.34, − 0.20)^*^
FEF_75_ (mL/s)	**− 22.70 (− 43.25, − 2.15)** ^*^	− 27.21 (− 49.28, − 5.15)^*^
PEF (mL/s)	− 39.95 (− 88.94, 9.04)	− 50.95 (− 99.81, − 2.09)^*^
FeNO (ppb)	0.34 (− 0.82, 1.49)	0.03 (− 1.28, 1.34)
No prenatal exposure to ETS ( n = 789)		
FVC (mL)	− 5.97 (− 22.15, 10.21)	2.33 (− 9.54, 14.20)
FEV_1_ (mL)	− 7.79 (− 22.78, 7.19)	− 1.27 (− 12.38, 9.84)
FEV_1_/FVC (%)	− 0.21 (− 0.64, 0.22)	− 0.33 (− 0.79, 0.14)
FEF_75_ (mL/s)	− 18.52 (− 37.62, 0.57)	− 16.11 (− 35.12, 2.90)
PEF (mL/s)	− 13.42 (− 56.35, 29.5)	0.82 (− 39.22, 40.87)
FeNO (ppb)	− 0.63 (− 2.02, 0.76)	− 0.70 (− 2.18, 0.78)

*CI* confidence interval, *ETS* environmental tobacco smoke, *FVC* forced vital capacity, *FEV*_1_ forced expiratory volume in 1 s, *FEF*_75_ forced expiratory flow at 75% of FVC, *PEF* peak expiratory flow; ppb: parts per billion

^a^Per 5-year increase in paternal age at birth, adjusting for age, sex, height, maternal age at birth, prematurity, birth weight, cesarean delivery, birth order, physician-diagnosed asthma, parental university education, parental allergic diseases, breastfeeding, and household income

^*^P < 0.05 is bold

**Table 4:** Association between paternal age (continuous, per 5-year increase) and lung function, stratified by breastfeeding

	Crude coefficient β (95% CI)^a^	Adjusted coefficient β (95% CI)^a^
Breastfed ≥ 6 months ( *n* = 611)		
FVC (mL)	− 11.99 (− 30.84, 6.85)	− 8.75 (− 23.34, 5.83)
FEV_1_ (mL)	− 14.79 (− 32.24, 2.65)	− 11.06 (− 24.42, 2.29)
FEV_1_/FVC (%)	− 0.35 (− 0.88, 0.17)	− 0.37 (− 0.95, 0.21)
FEF_75_ (mL/s)	− 21.10 (− 43.2, 1.00)	− 16.90 (− 39.49, 5.68)
PEF (mL/s)	− 14.17 (− 65.51, 37.17)	2.69 (− 46.8, 52.17)
FeNO (ppb)	0.69 (− 0.75, 2.13)	0.93 (− 0.67, 2.54)
Not breastfed or breastfed < 6 months ( *n* = 719)		
FVC (mL)	5.45 (− 10.24, 21.13)	4.31 (− 7.84, 16.47)
FEV_1_ (mL)	− 0.68 (− 14.89, 13.52)	− 3.20 (− 14.58, 8.17)
FEV_1_/FVC (%)	− 0.46 (− 0.86, − 0.06)^*^	− 0.60 (− 1.04, − 0.15)^*^
FEF_75_ (mL/s)	− 19.48 (− 36.80, − 2.16)^*^	− 22.31 (− 40.75, − 3.87)^*^
PEF (mL/s)	− 22.87 (− 62.41, 16.68)	− 29.94 (− 69.09, 9.2)
FeNO (ppb)	− 0.54 (− 1.73, 0.65)	− 1.29 (− 2.58, 0.00)

*CI* confidence interval, *FVC* forced vital capacity, *FEV*_1_ forced expiratory volume in 1 s, *FEF*_75_ forced expiratory flow at 75% of FVC, *PEF* peak expiratory flow, *ppb* parts per billion

^a^Per 5-year increase in paternal age at birth, adjusting for age, sex, height, maternal age at birth, prematurity, birth weight, cesarean delivery, birth order, physician-diagnosed asthma, parental university education, parental allergic diseases, prenatal exposure to environmental tobacco smoke, and household income

^*^P < 0.05 is bold

Table 5 shows the results treating paternal age at birth as a categorical variable. Negative associations were observed between paternal age at birth and the childhood FEV_1_/FVC and FEF_75_. Comparing to the reference group (paternal age at birth < 30 years), FEV_1_/FVC decreased 1.15% (95% CI − 2.20 to − 0.09), 1.20% (95% CI − 2.33 to − 0.08) and 2.93% (95% CI − 4.38 to − 1.47) in the groups of paternal age at birth 30–34, 35–39 and ≥ 40 years, respectively; FEF_75_ decreased 50.78 mL/s (95% CI − 95.95 to − 5.61) and 99.08 mL/s (95% CI − 157.66 to − 40.5), respectively, in the groups of paternal age at birth 35–39 and ≥ 40 years, with adjustment of relevant confounders (all *p* < 0.05).

**Table 5:** Association between paternal age at birth (categorical) and lung function

	Crude coefficient β (95% CI)^a^			Adjusted coefficient β (95% CI)^a^		
	**30–34 years (** ***n*** **= 566)**	**35–39 years (** ***n*** **= 398)**	**≥ 40 years (** ***n*** **= 159)**	**30–34 years (** ***n*** **= 566)**	**35–39 years (** ***n*** **= 398)**	**≥ 40 years (** ***n*** **= 159)**
FVC (mL)	20.51 (− 17.36, 58.37)	− 19.17 (− 59.10, 20.77)	9.17 (− 40.16, 58.50)	15.32 (− 12.17, 42.80)	− 16.50 (− 45.9, 12.9)	16.49 (− 21.64, 54.62)
FEV_1_ (mL)	4.62 (− 30.02, 39.25)	− 30.02 (− 66.55, 6.51)	− 21.20 (− 66.32, 23.92)	− 1.16 (− 26.69, 24.37)	**− 28.56 (− 55.87, − 1.24)** ^*^	− 20.50 (− 55.93, 14.93)
FEV_1_/FVC (%)	− 1.06 (− 2.06, − 0.06)^*^	− 1.15 (− 2.21, − 0.09)^*^	**− 2.29 (− 3.60, − 0.98)** ^*^	**− 1.15 (− 2.20, − 0.09)** ^*^	− 1.20 (− 2.33, − 0.08)^*^	− 2.93 (− 4.38, − 1.47)^*^
FEF_75_ (mL/s)	− 34.55 (− 77.52, 8.43)	**− 55.56 (− 100.88, − 10.24)** ^*^	**− 92.81 (− 148.80, − 36.83)** ^*^	− 34.11 (− 76.33, 8.11)	− 50.78 (− 95.95, − 5.61)^*^	− 99.08 (− 157.66, − 40.5)^*^
PEF (mL/s)	− 43.32 (− 142.24, 55.61)	− 97.48 (− 201.80, 6.85)	− 124.87 (− 253.74, 4.01)	− 41.62 (− 132.31, 49.08)	− 88.73 (− 185.76, 8.30)	− 115.26 (− 241.11, 10.58)
FeNO (ppb)	1.29 (− 1.62, 4.20)	0.78 (− 2.29, 3.84)	0.63 (− 3.14, 4.40)	0.38 (− 2.64, 3.40)	0.06 (− 3.16, 3.28)	− 0.24 (− 4.40, 3.92)

*CI* confidence interval, *FVC* forced vital capacity, *FEV*_1_ forced expiratory volume in 1 s, *FEF*_75_ forced expiratory flow at 75% of FVC, *PEF* peak expiratory flow, *ppb* parts per billion

^a^Reference group was paternal age at birth < 30 years, adjusting for age, sex, height, maternal age at birth, prematurity, birth weight, cesarean delivery, birth order, physician-diagnosed asthma, parental university education, parental allergic diseases, prenatal exposure to environmental tobacco smoke, breastfeeding, and household income

^*^P < 0.05 is bold

## Discussion

This study of 1330 children in a prospective population-based cohort is, to the best of our knowledge, the first to investigate the relationship between paternal age at birth and lung function and FeNO in offspring. Advanced paternal age at birth was significantly associated with decreasing FEV_1_/FVC ratio and FEF_75_ in offspring at school age. The findings remain significant after adjustment for pertinent factors, including age, sex, height, maternal age, prematurity, birth weight, cesarean delivery, birth order, asthma, parental university education, parental allergic diseases, prenatal exposure to ETS, breastfeeding, and household income. Furthermore, the negative association between paternal age at birth and childhood lung function was more pronounced among children with prenatal exposure to ETS or those who were not breastfed for the first 6 months.

Our results indicate adverse effect of advanced paternal age at birth on lung function in school-aged children, which is a novel finding from this study. Specifically, paternal age at birth equal to or over 40 years is significantly associated with 2.93% decrease in FEV_1_/FVC ratio and 99.08 mL/s decrease in FEF_75_. It is intriguing to note that the effect of advanced paternal age was found for FEV_1_/FVC ratio and FEF_75_, rather than FVC, suggesting an effect on large and small airway obstruction. The association of paternal age with lung function, but not with FeNO, suggests that advanced paternal age may contribute to airway structural change during early life, probably via a mechanism independent of allergic airway inflammation. However, the underlying biological mechanisms related to the identified associations between paternal age and lung function remain largely speculative. Several plausible explanations are provided as follows. First, adverse effects of paternal age at conception on offspring’s health outcomes may be related to de novo mutations, epigenetic alterations and/or fetal programing [7, 30]. Kong et al. found an association between father’s age and mutation rate in offspring, with an estimated effect of approximately two extra mutations per year corresponding to mutations doubling every 16.5 years [31]. It might likely lead to adverse impact on health outcomes. Second, paternal age has been shown to play a role in the vertical transmission of telomere length [32–34]. Previous reports have provided evidence that lung function in children and adults is associated with telomere length [35–38]. Therefore, it may be speculated that advanced paternal age may affect offspring’s lung function through alterations of telomere length. Further investigation is merited to scrutinize underlying biological mechanisms related to adverse effects of paternal age on offspring’s lung function.

The impact of parental age on lung function in offspring is largely unclear. At a first glance, the finding of lower lung function with increasing paternal age in this study may seem contradictory to an European study showing higher lung function related to increasing maternal age [12]. Although maternal ageing in relation to higher lung function in the previous study is rather contra-intuitive [12], it might remain possible that paternal age and maternal age could have different biological effects on offspring’s respiratory health. That is, gender difference might have played a role on modulating different effects of paternal age and maternal age on respiratory health in offspring. However, underlying regulatory mechanisms are currently unknown. This difference therefore calls for further research on better understanding of biological mechanisms related to parental ageing effects on respiratory outcomes in offspring.

Our findings suggest that children with prenatal exposure to ETS or not being breastfed are more vulnerable to adverse effect of advanced paternal age on childhood lung function. There is clear evidence linking exposure to tobacco smoke during the perinatal period with impaired lung function in children [24, 25]. Our study provides additional evidence for a synergistic adverse effect of prenatal exposure to ETS and advanced paternal age on the lung function in offspring. Several studies have shown that breastfeeding could improve lung function in children [26–28]. Our study suggests that breast milk may more effectively promote the programming of the children’s developing respiratory system than infant formula and counteract the harmful effect of advanced paternal age.

The strengths of this study included a large population-based cohort of children and measurement of lung function and FeNO as objective markers of respiratory health. However, there are some limitations. First, it remains possible that the observed associations might be partly explained by unmeasured confounding factors, although this study has adjusted relevant factors in the analyses. Second, whether the current findings in Asian children could be generalized to other populations need further confirmation.

In conclusion, this large population-based cohort study provides new evidence linking advanced paternal age with decreasing lung function at school age. Children with prenatal exposure to ETS or not being breastfed are more vulnerable to adverse effect of advanced paternal age on childhood lung function. Further studies are warranted to confirm this novel adverse effect of advanced paternal age.

## Supplementary information

**Additional file 1: Table S1. **Association between paternal age at birth(continuous, per 5-year increase) and lung function (converting to z-scores). 

## Data Availability

Not applicable.

## Abbreviations

FeNO: Fraction of exhaled nitric oxide
LIGHTS: Longitudinal Investigation of Global Health in Taiwanese Schoolchildren
ATS: American Thoracic Society
ERS: European Respiratory Society
FVC: Forced vital capacity
FEV_1_: Forced expiratory volume in 1 s
FEF_75_: Forced expiratory flow at 75% of forced vital capacity
PEF: Peak expiratory flow
ETS: Environmental tobacco smoke
SD: Standard deviation
NTD: New Taiwan dollar
ppb: Parts per billion
