# Supplementary material for: Association of advanced paternal age with lung function at school age
Source: Respir Res. 2022 Sep 20;23:259. doi: 10.1186/s12931-022-02178-4 (PMC9487029; doi:10.1186/s12931-022-02178-4)
Supplement: Supplementary file 1 — Additional file 1: Table S1. Association between paternal age at birth(continuous, per 5-year increase) and lung function (converting to z-scores). [file 12931_2022_2178_MOESM1_ESM.docx]

**SUPPLEMENTAL APPENDIX**

**Table S1.** Association between paternal age at birth (continuous, per 5-year increase) and lung function (converting to z-scores).

|  | **Crude coefficient β (95% CI)^a^** | **Adjusted coefficient β (95% CI)^a^** |
| --- | --- | --- |
| FVC z-score | 0.01 (-0.04, 0.06) | 0.00 (-0.05, 0.06) |
| FEV_1_ z-score | -0.02 (-0.07, 0.03) | -0.04 (-0.09, 0.02) |
| FEV_1_/FVC z-score | **-0.08 (-0.14, -0.03)^*^** | **-0.10 (-0.16, -0.03)^*^** |
| FEF_75_ z-score | **-0.07 (-0.12, -0.02)^*^** | **-0.08 (-0.13, -0.02)^*^** |
| PEF z-score | NA^b^ | NA^b^ |

CI: confidence interval; FVC: forced vital capacity; FEV_1_: forced expiratory volume in 1 second; FEF_75_: forced expiratory flow at 75% of FVC; PEF: peak expiratory flow; ppb: parts per billion.

^a^ Per 5-year increase in paternal age at birth, adjusting for age, sex, height, maternal age at birth, prematurity, birth weight, cesarean delivery, birth order, physician-diagnosed asthma, parental university education, parental allergic diseases, prenatal exposure to environmental tobacco smoke, breastfeeding, and household income.

^b^ Reference values for PEF were not available in the Global Lung Function Initiative 2012 reference equations.

**^*^** *P* <0.05 is bold.
